# Attention Deficit Hyperactivity Disorder (ADHD) and Polyphenols: A Systematic Review

**DOI:** 10.3390/ijms25031536

**Published:** 2024-01-26

**Authors:** Fabrizio Turiaco, Chiara Cullotta, Federica Mannino, Antonio Bruno, Francesco Squadrito, Giovanni Pallio, Natasha Irrera

**Affiliations:** 1 Department of Clinical and Experimental Medicine, University of Messina, 98125 Messina, Italy; fabrizio.turiaco@gmail.com (F.T.); chiara.cullotta@studenti.unime.it (C.C.); fmannino@unime.it (F.M.); fsquadrito@unime.it (F.S.); nirrera@unime.it (N.I.); 2 Department of Biomedical and Dental Sciences and Morphological and Functional Imaging, University of Messina, 98125 Messina, Italy; antonio.bruno@unime.it

**Keywords:** polyphenols, oxidative stress, neuroinflammation, ADHD

## Abstract

Polyphenols are natural compounds also contained in daily consumed foods that show their efficacy in different clinical fields. Both pre-clinical and clinical studies demonstrated that polyphenols may manage neuroinflammation and oxidative stress processes tightly connected to neurodegenerative diseases and mental disorders. Thus, a neuroinflammatory state may influence the neurotransmitters pathways, such as the noradrenergic, glutamatergic, serotoninergic, and, in particular, dopaminergic ones, whose impairment is strongly associated with attention deficit hyperactivity disorder (ADHD). Therefore, the aim of the present systematic review is to provide an overview of the clinical outcomes’ changes following ADHD treatment with polyphenols alone and in combination with the traditional drugs. This review was conducted according to PRISMA guidelines and recorded on PROSPERO with the number CRD42023438491; PubMed, Scopus, and Web of Science were used as search-engines to lead our research until June 2023. The inclusion criteria were articles written in English, including clinical, placebo-controlled, and case-control trials. We excluded reviews, metanalyses, background articles, and papers published in other languages. To avoid any bias, Rayyan software (COPYRIGHT © 2022 RAYYAN) was used to organize the work and manage the literature review. After screening, 10 studies were included, with a total of 556 patients that met the established inclusion criteria. The data obtained from these studies showed that polyphenols rebalanced oxidative stress pathways through different mechanisms, are effective for the treatment of ADHD both alone and in combination with traditional drugs, and are able to reduce symptoms as well as the side effects related to the use of conventional therapies. Finally, a positive effect of using polyphenols for ADHD prevention could be hypothesized.

## 1. Introduction

Polyphenols are natural compounds found in plants, stored as glycoside and non-glycosylated conjugates, that show several positive effects in human health. These natural products are heterogeneous and often share their chemical structure as well as their origin and biological activities by allowing for a classification in flavonoids, phenolic acids, stilbenes, lignans, and tannins [1]. Polyphenols are also contained in daily consumed foods, but their absorption is irregular and may be influenced by different factors, including the dietary fiber consumption and microbiome. Therefore, their bioavailability is extremely variable, affecting their biological effects, and represents the main limitation of using polyphenols as a therapeutic approach [2,3].

The main effect of polyphenols is related to the modulation of inflammation and oxidative stress mechanisms that play a critical role in the pathogenesis of different diseases, as well as in those affecting the nervous system [4]. In this context, neuroinflammation triggered by pathogen-associated molecular patterns (PAMPs) and/or damage-associated molecular patterns (DAMPs) may induce neuronal loss by stimulating cell death [4]. The activation of cell death mechanisms may also be the consequence of the activation of the pro-inflammatory nuclear factor-kappa B (NF-κB) and the increase in cytokines whose release is dependent on activated microglia, reactive astrocytes, and infiltrating peripheral immune cells [5,6]. Also, oxidative stress imbalance may cause cell death since reactive oxygen species (ROS) induce oxidative damage to lipids, proteins, and DNA, as well as mitochondrial function impairment, thus leading to immune cells activation. Under physiological conditions, ROS could be inactivated by the antioxidant effectors, including the enzymes catalase (CAT) or glutathione peroxidase, with a consequent reduction in glutathione (GSH) levels; however, an impairment in redox homeostasis can occur when GSH levels decrease in favor of oxidized glutathione (GSSG) [7]. Moreover, the failure of redox homeostasis activates cytoprotective molecular pathways, which also upregulate antioxidant proteins, such as the nuclear factor erythroid 2-related factor 2 (Nrf2). In particular, Nrf2 is mostly located in the cytosol interacting with Kelch-like ECH-associated protein 1 (Keap1); redox imbalance causes the detachment of Nrf2 from Keap1, with the resulting translocation into the nucleus. Nrf2 acts as a transcriptional activator for the genes strictly involved in redox homeostasis, like antioxidant proteins and enzymes involved in metabolizing xenobiotics and proteins [8]. Previous studies indicated that natural compounds could improve the oxidative stress imbalance; in particular, Chlorogenic Acid (CGA, 3-CQA), a biologically active phenolic acid contained in different foods, such as artichoke and green coffee bean [9], was able to induce an increase in the anti-oxidant Nrf2 and a reduction in the pro-inflammatory transcription factor NF-κB [10] as well as the pro-inflammatory markers Prostaglandin E_2_ (PGE_2_), Interlukin-1 β (IL-1β), Tumor Necrosis Factor α (TNF-α), and Cyclooxygenase-2 (COX-2) [11], thus showing significant anti-oxidant and anti-inflammatory effects. Also, Resveratrol, a stilbenoid polyphenol found in grapes, nuts, and berries, was able to modulate neuroinflammation (thanks to COX and NF-κB inhibition) and to downregulate cell death and oxidative mechanisms in order to preserve neuronal loss [12,13,14]. Recently, it has been demonstrated that Epigallocatechin-3-gallate, one of the most abundant metabolites of Pine Bark Extract (PBE), might stimulate ROS clearance and inhibit kinases driving NF-κB gene expression [15]. In fact, PBE antioxidant and anti-inflammatory properties were demonstrated, generally with a formulation known as Pycnogenol^®^, which is a polyphenol-rich extract, standardized to contain 70% of oligomeric procyanidins, such as catechin, epicatechin, taxifolin, caffeic acid, and ferulic acid. All these compounds are involved in the biological defense from ROS and inflammation typically recognized at the biological basis of psychiatric disorders. Among the wide range of psychiatric diseases, recent findings suggested that ADHD is highly associated with redox imbalance [16]. In fact, the lack of antioxidant barriers and the increase in oxidative stress and inflammatory processes represent significant risk factors for the pathophysiology of different psychiatric disorders, including attention deficit hyperactivity disorder (ADHD) [16]. ADHD is a psychiatric disorder that affects males and females (2:1 ratio) [17], with a childhood onset generally before the age of 7, although a late onset up to 12 years could also be observed, with a prevalence of about 5% in school-aged children and 2.5% in adulthood [18]. Neuroinflammation together with glial activation, oxidative stress, and reduced neurotrophic support affect brain development and neurotransmitter function, thus increasing the risk of the appearance of neurodevelopmental disorders [19]. In fact, the neuroinflammatory state may influence many neurotransmitter pathways such as the dopaminergic, noradrenergic, glutamatergic, and serotoninergic ones. The alteration of dopamine levels is strongly associated with ADHD. In fact, ADHD patients show reduced dopamine synthesis, insufficient production, and the release of dopaminergic synaptic vesicles, with the consequent inhibition of dopamine receptors activity and increased dopamine reuptake degradation [20]. For this reason, methylphenidate is one of the most used drugs that acts as an indirect agonist of dopaminergic synapses. Even alterations of the dopamine transporter and dopamine receptor genes have been associated with ADHD. Dopamine receptors belong to G-protein coupled receptors (GPCRs) that can be grouped into two classes: D1-like (D1 and D5) and D2-like (D2, 3, and 4); polymorphisms of D4 and D5 receptor coding genes have been associated with ADHD [21].

This review aims at evaluating polyphenols’ anti-inflammatory and antioxidant effects in order to assess their possible use as innovative therapeutic strategies for the modulation of neuroinflammation and in particular for the prevention and treatment of ADHD. In fact, traditional drugs used for ADHD treatment may be responsible for different adverse effects, such as hyporexia (25% of patients), xerostomia (20%), headache (20%), nausea (10%), and insomnia (10%) [22]. Polyphenols could be useful in reducing the side effects related to the therapy and could also be administered in association with traditional psychotropic drugs (amphetamine, methylphenidate, atomoxetine, bupropion) to enhance their effect. Moreover, patients often show poor compliance with the pharmacologic treatment; therefore, the development of a treatment based on the use of natural products could be easily accepted by patients that are unconfident with pharmacological treatment, bringing benefits to the quality of healthcare and the quality of life of patients.

## 2. Materials and Methods

### 2.1. Search Strategy

This review was conducted according to PRISMA (Preferred Reporting Items for Systematic Reviews and Meta-Analyses) guidelines [23] and recorded on PROSPERO with the number CRD42023438491. PubMed, Scopus, and Web of Science were used as search-engines in order to evaluate the state of the art up to June 2023. The following search string was applied for all the search-engines: ((adhd OR “attention deficit hyperactivity disorder”) AND polyphenol*).

### 2.2. Inclusion and Exclusion Criteria

A first selection was independently performed by two researchers reading titles and abstracts, according to the following inclusion criteria: articles written in English and containing quantitative and qualitative information about ADHD and polyphenols, including clinical trials, placebo-controlled trials, and case-control trials. The exclusion criteria were the following: articles published in other languages, reviews, meta-analyses, background articles, and articles not relevant to the topic.

### 2.3. Extraction of Relevant Data, Quality, and Risk of Bias Assesment

Relevant data were extracted and compared through a data extraction sheet. The extraction procedure was conducted by FT and CC. The extracted data included the (I) study design, (II) number of patients, (III) polyphenols studied, and (IV) main results. To prevent any bias, Rayyan software (COPYRIGHT © 2022 RAYYAN) (https://www.rayyan.ai) was used to organize the work and manage the literature review.

To assess the risk of bias in the findings, we used RoB version 2.0, the revised Cochrane risk of bias tool for randomized trials. RoB assesses the following domains: the randomization process, deviations from intended interventions, missing outcome data, the measurement of the outcome, and the selection of the reported results. As for the study selection, two individuals independently estimated the risk of bias for each study. Disagreements have been resolved by consensus between the authors or by a third author. No significant publication bias was detected among the articles. Table 1 shows the risk of bias assessment.

## 3. Results and Discussion

**Figure 1:**
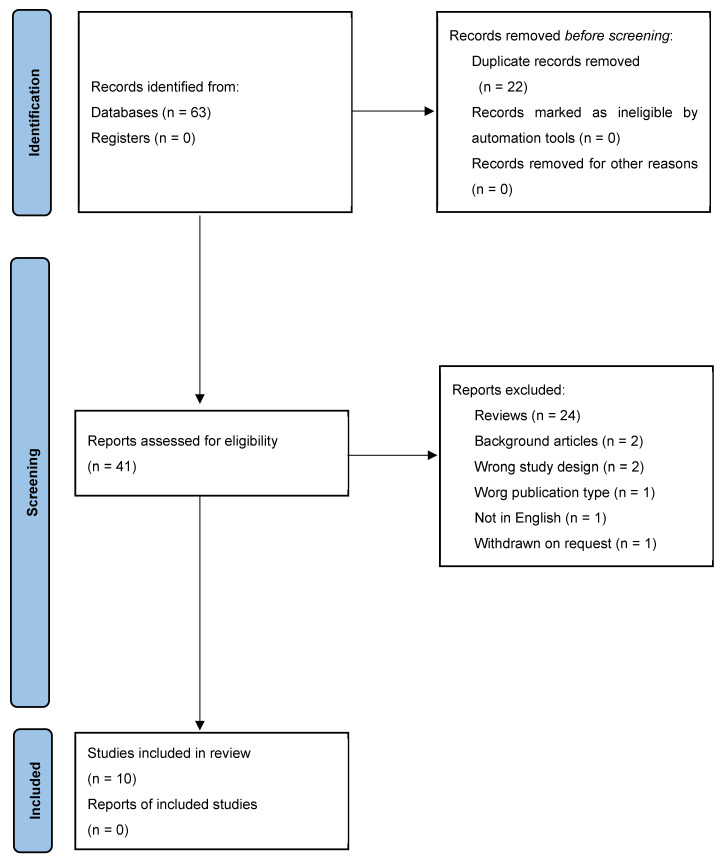
Flow diagram of the literature selection process.

Figure 1 shows the flow chart describing the process of the articles selection for the present Systematic Review. In the preliminary search, 14 articles on PubMed, 19 on Scopus, and 30 on Web of Science were found.

From the total of 63 research papers, 41 articles were selected after the removal of duplicates (*n* = 22). Of these 41 papers, after the title and abstract evaluation by two independent researchers, 24 studies were excluded because they were reviews, 2 were excluded because they were background articles, 2 were excluded because of a wrong study design, 1 was excluded because of a wrong publication type, 1 was excluded because the language was not English, and 1 was excluded because it was withdrawn at the request of the authors and/or editor. Following this screening, we found 10 papers that evaluated polyphenols’ effects on patients affected by ADHD and who were included in the final review.

### 3.1. Efficacy on ADHD Symptoms: Polyphenols vs. Traditional Drugs and Placebo

From our research, we identified 5 studies that evaluated polyphenols’ efficacy on ADHD symptoms. In particular, in the study of Weyns et al., 88 pediatric patients (aged 6 to 12 years) affected by ADHD were randomized 1:1:1 to receive methylphenidate (MPH; 20 or 30 mg/day if < or ≥30 kg), Pycnogenol^®^ (PBE; 20 or 40 mg/day if < or ≥30 kg), or placebo. The participants were comparable for baseline characteristics, and all outcomes were evaluated at baseline, 5 weeks, and 10 weeks following treatment. ADHD-RS, which is a teacher-report and parent-report inventory used for the diagnosis of ADHD, was used to evaluate the clinical effects of PBE and MPH.

Significant differences were observed in the summed score of the teacher and parent ADHD-Rating Scale (ADHD-RS), and the mean total scores were 26.07 and 18.43 in the PBE group, 24.77 and 13.50 in patients treated with MPH, and 30.06 and 28.60 in the placebo group at baseline and after 10 weeks of treatment, respectively. However, no differences were found between MPH and PBE following post hoc analyses. Therefore, the drug effects differed from those of the placebo for the total score (MPH vs. placebo *p* < 0.01 and PBE vs. placebo *p <* 0.05) and for hyperactivity/impulsivity score (MPH vs. placebo *p <* 0.05 and PBE vs. placebo *p <* 0.01), whereas only MPH showed significant differences compared to the placebo in the inattention score (*p <* 0.05 after 5 weeks and *p* < 0.01 after 10 weeks). Significant differences between treatments were found after 10 weeks in the parent-rated summed ADHD-RS and inattention sub-scores. No differences emerged between MPH and PBE following post hoc analyses; MPH effects significantly differ from those of the placebo in the total score (*p <* 0.01), hyperactivity/impulsivity (*p <* 0.05), and inattention sub scores (*p <* 0.01).

The Parent Socio-Emotional Questionary (SEQ), which is an effective psychiatric screening method for hyperactivity and emotional disorders, showed significant differences between the MPH and placebo groups (*p <* 0.05) and between the PBE and placebo groups (*p <* 0.01) in the total score. A significant difference was found in the hyperactivity sub-score between MPH and the placebo (*p* < 0.01) and between PBE and MPH (*p <* 0.001). Post hoc analyses on teacher ratings revealed that the percentage of treatment responders significantly differs between MPH and the placebo (*p* = 0.007). Moreover, post hoc analyses revealed that a significant increase in the adverse events number was observed in MPH-treated patients compared to the PBE-treated group both after 5 and 10 weeks of treatment (*p* = 0.004 and *p* = 0.0255, respectively). The main adverse effects reported were headache, dizziness, nausea, and diarrhea following PBE treatment and gastrointestinal symptoms, reduced appetite, insomnia, headache, tachycardia, sneezing, and being emotional in MPH-treated patients [24].

Hsu et al. conducted a double-blinded randomized placebo-controlled cross-over study including 20 ADHD patients (17 males and 3 females, aged 10 years). The patients were randomly assigned to receive placebo or PBE during the first 4 weeks, and, after a washout period (weeks 5 and 6), another supplement was administered for an additional 4 weeks (weeks 7 to 10). In particular, one or two capsules (containing 25 mg PBE) were administered daily depending on the body weight (≤50 kg or >50 kg). A significant reduction in the percentile rank of the inattention item was observed in the PBE-treated patients compared to the baseline (*p* = 0.023). A significant reduction was also observed in the hyperactivity-impulsivity parameter after PBE supplementation compared to the baseline (*p* = 0.029) and compared to the placebo (*p* = 0.024). Moreover, the evaluation of inattention and impulsivity through the Conners Continuous Performance Test 3rd edition (CPT-III) revealed that the T-score of commissions was significantly reduced compared to the baseline after 4 weeks of PBE supplementation (*p* = 0.048) [25].

Trebatická et al. designed a randomized, double-blind, placebo-controlled study including 61 pediatric patients (50 boys and 11 girls), who were randomized 2.5:1 to receive Pycnogenol^®^ (1 mg/kg body weight) or placebo and were evaluated at time 0 (baseline), at time 1 (one month of treatment), and at time 2 (after one month of wash-out). Among teachers’ psychodiagnostic tools, the Child Attention Problem (CAP) teacher rating scale revealed no significant differences between the groups at time 0 for hyperactivity as well as for inattention. At time 1, after treatment with Pycnogenol^®^, the scores for hyperactivity and for inattention significantly dropped compared to those at the beginning of the study (*p* = 0.008 and *p* = 0.00014, respectively) and compared to the placebo (*p* = 0.044 and *p* = 0.0067, respectively). At time 2, after the wash-out, the ADHD symptoms scores came back to the same level of the beginning.

Conner’s Teacher rating Scale (CTRS) showed a significant reduction in inattention compared to the beginning (*p* = 0.07) and compared to the placebo (*p* = 0.049), and the score for hyperactivity of Conner’s Parent Rating Scale (CPRS) decreased with a marginal significance compared to the placebo after 1 month of treatment with Pycnogenol^®^ (*p* = 0.065).

The weight scores of tests for visual-motoric coordination and concentration were different for the placebo and Pycnogenol^®^ groups at the beginning; therefore the changes after treatment were evaluated as percentage changes relative to the beginning. A significant percentage increase was observed in the weight scores following 1 month of Pycnogenol^®^ treatment compared to the beginning of treatment (*p* = 0.019) and compared to the placebo group (*p* = 0.05) [26]; this side effect was due to the increased production of neuropeptide Y [34]. The only study showing the opposite trend was the double-blind, placebo-controlled, crossover study of Tenenbaum et al., in which every participant received a 3-week administration of methylphenidate, Pycnogenol^®^, and placebo, separated by a week of wash-out between every treatment, without any results being statistically significant for all treatments investigated and outcomes evaluated [27].

In the 8-week, randomized, double-blind, placebo-control trial conducted by Rafeiy-Torghabeh et al., patients were treated with Methylphenidate for the first and second week of the trial, at a dose of 10 mg/day and 20 mg/day depending on the body weight; a dose of 20 mg/day was then maintained from week 3 until the end of the trial, although patients who weighed more than 30 kg were treated with a maintenance dose of 30 mg/day. One group of patients received Resveratrol (ACER, 500 mg/day) as an add-on to MPH, while the other group received 500 mg/day of starch as a placebo. To evaluate ADHD symptoms, teacher and parent ADHD-RS-IV values were used. Only parent ADHD-RS showed significant results. At baseline, the total scores of parents’ ADHD-RS-IV were 33.93 for the resveratrol group and 34.10 for the placebo. At week 4, the scores decreased to 10.27 and 15.50, respectively, whereas at week 8, they reached 8.50 and 10.87, respectively, (*p* = 0.015). The inattention sub-scores were 16.33 and 16.20 at baseline, 5.47 and 7.53 at week 4, and 4.50 and 4.93 at week 8 in the resveratrol and placebo groups, respectively, with *p* = 0.032. Finally, the hyperactivity sub-scores were 17.60 and 17.90 at baseline, 4.80 and 7.97 at week 4, and 4.00 and 5.93 at week 8 in the resveratrol and placebo groups, respectively (*p* = 0.036) [28].

In summary, by exploiting different approaches, Weyns et al., Hsu et al., and Trebatická et al. reported that patients treated with Pycnogenol showed an improvement in ADHD symptoms comparable to that observed following methylphenide treatment. The only opposite finding was detected by Tenenbaum and colleagues, who noticed no significant effects in the PBE group compared to the placebo. Finally, concerning the use of resveratrol as an add-on to methylphenidate, Rafeiy-Torghabeh et al. showed significant results by revealing a decrease in the total scores and the inattention and hyperactivity sub-scores [24,25,26,27,28]; Table 2.

### 3.2. Efficacy of Polyphenols in Rebalancing Oxidative Stress Pathways

The study of Weyns and colleagues (Part 2) also focused on oxidative stress/antioxidant pathways in the same cohort of 88 pediatric patients. No significant differences were observed in GSH levels between treatments, as for α-tocopherol, γ-tocopherol, β-carotene, retinol, and coQ10. On the other hand, a significant difference was found in CAT activity after 10 weeks (*p* = 0.025), although it has been hypothesized that this result could be a false positive. Glutathione peroxidase (GPX), catalase (CAT), superoxide dismutase (SOD), xanthine oxidase (XO), and Apolipoprotein J (*ApoJ*) gene expression did not reveal any significant difference between the groups [29].

In the study of Hsu et al., PBE antioxidant effects were also investigated in children with ADHD through the GSH/GSSH ratio, 2-thiobarbituric acid-reactive substance (TBARS), and 8-isoprostane levels. After 4 weeks of PBE supplementation, an improvement in the antioxidant status was observed due its antioxidant effects; in fact, the erythrocytic GSH/GSSG ratio was significantly increased compared to the baseline (*p* = 0.0358) and placebo supplementation (*p <* 0.0336). Also, TBARS, which is an index of lipid peroxidation, was significantly reduced following PBE supplementation (*p* = 0.0026 vs. placebo). In addition, the sodium level was significantly lower than those observed at baseline following PBE supplementation, whereas aspartate aminotransferase (AST) and alanine transaminase (ALT) activity and blood urea nitrogen (BUN) levels were not reduced [25].

A double-blinded randomized placebo-controlled study was conducted by Dvořáková and colleagues with the participation of 43 ADHD out-patients (34 boys and 9 girls, aged from 6 to 14 years) that received Pycnogenol^®^ (1 mg/kg body weight) or a placebo. Patients with other psychiatric disorders (mood, anxiety, personality disorder, or personality changes due to a general medical condition) and patients with acute inflammatory diseases, renal and cardiovascular disorders, and diabetes were excluded.

All participants were evaluated for bilirubin, glucose, γ-glutamyl transferase, alkaline phosphatase, aspartate aminotransferase, alanine aminotransferase, uric acid, and lipid profiles: the baseline measurements were within the normal and expected ranges, and any variation beyond the normal range was observed after 1 month of Pycnogenol^®^ or placebo administration.

In particular, parameters reflecting redox homeostasis, including GSH, GSSG, and total antioxidant status (TAS), were evaluated. A significant reduction was observed in GSSG levels, with a percentage decrease of 22.03% in the Pycnogenol^®^ group but not in the placebo group after one month of administration; in particular, GSSG values changed from 4.60 μmol/L to 3.58 μmol/L (*p* = 0.013). However, GSSG levels raised again following a wash-out period (one month) in the Pycnogenol^®^ group. Patients treated with Pycnogenol^®^ showed GSH values of 102.89 μmol/L at the beginning of the study that significantly increased (26.8%) after 1 month (*p* = 0.0054); this gain (36.4%) also persisted in the period of the investigation after the washout period in patients receiving Pycnogenol^®^ (*p* = 0.007). The placebo did not affect GSH levels or GSSG. Moreover, an increase in TAS levels was observed in the Pycnogenol^®^-treated group, although these growths were not significant compared to those detected at the beginning of the study. Conversely, a significant difference was recorded after the wash-out period (*p* = 0.002). No significant change was observed in TAS levels in the group treated with the placebo. A negative correlation between TAS and GSSG levels was detected in the Pycnogenol^®^ group (*p* < 0.05), whereas a positive correlation between the inattention score and TAS was found (*p* = 0.035). No correlation was found in the placebo group [30].

The management of hyperactivity in children through catecholamine excretion and oxidative stress regulation has been hypothesized in another study by Dvořáková and colleagues (2007) that evaluated both the overactivity of the adrenergic system and the oxidative capacity of blood (GSH/GSSG ratio) by measuring levels of Adrenaline (A), Noradrenaline (NA), dopamine (D), and both GSSG and GSH levels. In this study, 47 boys and 10 girls with ADHD, aged between 6 and 14 years, were enrolled. They were supplemented with Pycnogenol^®^ (PBE) daily at a dose of 1 mg/kg or placebo for one month. Children suffering from ADHD showed a significant increase in A (10.13 nmol/mmol of creatinine (nmol/mmol crea) *p* = 0.001) and NA (22.51 nmol/mmol crea *p* = 0.007) levels compared to healthy children (2.09 nmol/mmol crea for A and 15.55 1.06 nmol/mmol crea for NA) at baseline, whereas urinary concentrations of D were similar in both groups. The urinary concentrations of all monoamines decreased following PBE supplementation. In particular, the decrease in the D concentration (nmol/mmol crea) average from 226.1 to 202.4 was significant (*p* < 0.05) in comparison with the values of the PL-treated group (211.0 vs. 206.2, *p* = 0.88). The decline (from 10.3 to 7.6 nmol/mmol crea) was about 26.2%, and the NA reduction (from 24.1 to 19.9 nmol/mmol crea) was about 17.1%, thus reaching non-significant values. No changes in catecholamine levels were found in response to the PL treatment. The concentrations of dopamine, adrenaline, and noradrenaline were higher in PBE than in PL-treated groups, even if any difference was observed after the wash-out period (one month). No significant correlation was found in the PL group, but a positive correlation (*p* = 0.031) was observed between NA levels and the hyperactivity of children with ADHD before the trial. Moreover, a positive correlation between dopaminergic overactivity and oxidized glutathione was detected. The calculated GSH/GSSG ratio was 35.93 in patients with ADHD at the beginning of the trial; after PBE treatment, the GSH/GSSG ratio increased to 52.26 (*p* = 0.05) and decreased to 42.45, following one month of wash-out. No change was found in the GSH/GSSG ratio in the PL group. The observed changes represent a positive correlation between GSSG and adrenaline (*p* < 0.007) as well as noradrenaline (*p* < 0.003) levels before the trial. After PBE treatment, a negative correlation was obtained between the GSH/GSSG ratio and dopamine concentrations (*p* = 0.0147): these results finally demonstrated an improvement in redox homeostasis and in dopaminergic neurotransmission. However, after one month of PBE administration, no correlation was proven between catecholamine concentrations and GSSG levels [31].

Chovanová et al. tested the effect of a polyphenolic extract of pine bark (Pycnogenol^®^) on TAS and on the level of oxidized purines represented by 8-oxo-7,8-dihydroguanine (8-oxoG) in children with ADHD. Sixty-one patients (50 boys and 11 girls) with ADHD, aged between 6 and 14 years, were enrolled in this randomized, double-blind, placebo-controlled study. Basic biochemical parameters (bilirubin, glucose, gamma-glutamyl transferase, alkaline phosphatase, aspartate aminotransferase, alanine aminotransferase, uric acid, and lipid profile) were analyzed before the trial, 1 month after PBE (1 mg/kg body weight) or placebo administration, and after the wash-out period, and all values were in the physiological range. No association was found between 8-oxoG levels, used as a marker of oxidative stress, since ROS might modify the guanine or deoxyguanosine of nuclear and mitochondrial DNA and clinical parameters before the trial.

At the beginning of the trial, damages in DNA were detected by a comet assay and were significantly increased in patients with ADHD in comparison to healthy controls (198.5 vs. 61.7 total damage score, *p* < 0.001). The levels of 8-oxoG (0.412 8-oxoG/106 G) were significantly decreased after 1 month of PBE treatment in comparison to initial values (0.558 8-oxoG/106 G, *p* < 0.012) and to the placebo group (0.412 8-oxoG/106 G for PBE vs. 0.638 8-oxoG/106 G for placebo group, *p* < 0.014). No significant change in 8-oxoG levels was observed in the placebo group. After the wash-out period (1 month after the termination of PBE administration), the 8-oxoG level rose again. TAS was decreased in the plasma of children with ADHD (1.026 mM) when compared to the reference values (1.1–1.7 mM); after 1 month of PBE administration, TAS increased (1.050 mM) but without reaching significance (*p* = 0.119). Instead, a significant rise in the TAS level was found after the wash-out period in comparison with periods 1 and 0 (1.091 mM in period 2 vs. 1.026 ± 0.021 mM in period 0, *p* < 0.0019). Placebo administration had no effect on TAS, and a negative association between TAS and 8-oxoG (y = 20.082x + 1.069, r = 20.217, *n* = 54, *p* = 0.048) was found before the trial [32].

In summary, in the study conducted by Weyns et al., no significant difference in the GSH concentration was observed between treatments, as for α-tocopherol, γ-tocopherol, β-carotene, retinol, and coQ10. Instead, a significant difference was detected in the CAT activity between groups after 10 weeks, although this result could be considered as a false positive. In support of this evidence, neither *GPX*, *CAT, SOD*, *XO*, nor *ApoJ* gene expression revealed any significant difference between the groups.

These results could be due not only to the different doses of PBE used in this clinical trial compared to those administered in other studies (up to 100 mg/kg) but also to the highest oxidative stress levels in the animal models compared to those observed in ADHD patients. In addition, low levels of antioxidant enzymes do not necessarily correlate with increased oxidative stress: polyphenols may activate the Nfr2 pathway, thus inducing an increase in the glutathione-S-transferase (GST) cytoprotective gene, which could be responsible for lower GSH concentrations [29].

Hsu et al. found that the erythrocytic GSH/GSSG ratio significantly increased compared to the baseline and placebo supplementation after 4 weeks of PBE supplementation, and TBARS was significantly reduced compared to the placebo group. Dvořáková and colleagues demonstrated a significant decrease in GSSG levels and a significant increase in GSH levels after one month of treatment with Pycnogenol^®^. The GSH levels increase was also detected during the wash-out period, together with a growth of TAS levels that was positively correlated with the inattention score; second, the dopamine urinary concentration significantly decreased in comparison to the placebo group after 1 month of PBE treatment, whereas the GSH/GSSG ratio increased and then decreased following one month of wash-out, and a negative correlation between the GSH/GSSG ratio and dopamine concentrations was found, thus demonstrating an improvement in redox homeostasis and in dopaminergic neurotransmission. Chovanová et al. found that the levels of 8-oxoG significantly decreased after 1 month of PBE treatment in comparison to the initial values and to the placebo group, but after the wash-out period, the level of 8-oxoG rose again. TAS was decreased in the plasma of children with ADHD when compared to the reference values (1.1–1.7 mM); on the contrary, its level rose after the wash-out period in PBE-treated patients [25,29,30,31,32,33]; Table 3.

### 3.3. Polyphenols May Be Protective against ADHD

A case-control study, including 200 ADHD patient and 200 healthy controls, was conducted by Darzi et al. to evaluate the risk of ADHD per unit of polyphenol intake (mg/100 gr). A Food Frequency Questionnaire (FFQ), which is a 168-item semi-quantitative interview, constituted by a list of the most common food consumed by participants, was administered. An indirect relationship between dietary polyphenol consumption and ADHD risk was shown (OR: 0.995, 95% CI = 0.994 to 0.996, *p* < 0.001). The results remained significant even after the first correction adjusting for energy intake (OR: 0.995, 95% CI = 0.994 to 0.996, *p* < 0.001) and after a second correction adjusting for BMI, socioeconomic status, age, and gender (OR: 0.992, 95% CI = 0.989 to 0.995, *p* < 0.001).

In summary, Darzi et al., evaluating the risk of ADHD per unit (mg/100 gr) of polyphenol intake, found an indirect relationship between dietary polyphenol consumption and ADHD risk [33]; Table 4.

## 4. Conclusions

The effects of polyphenolic compounds were explored in the present Systematic Review in the context of ADHD prevention and treatment for the first time.

Polyphenols emerged as effective for ADHD treatment both alone and in combination (resveratrol) with methylphenidate, thus reducing the symptoms and adverse events appearance; only one study did not show evidence of effectiveness.

In particular, polyphenols were able to rebalance oxidative stress pathways through different mechanisms, GSH increase and GSSG reduction, by shifting the ratio in favor of the antioxidant effect; lipid peroxidation decrease and TAS levels restoration positively correlated with the decrease in inattention symptoms in ADHD patients. Moreover, polyphenols showed DNA damage reduction properties, demonstrated by 8-oxoG decrease, as well as the ability of catecholamine neurotransmission rebalance related to hyperactivity symptoms amelioration; finally, the evidence of a positive effect in ADHD prevention was highlighted. Therefore, the data described so far suggest a possible use of polyphenolic products for the management of ADHD, both in pediatric and adult clinical settings.

However, a further exploration and robust evidence are needed before making conclusive statements regarding the effective clinical potential of polyphenols.

## 5. Limits of the Study

The heterogeneity of the age of the examined population, mostly pediatric, represents one of the limits of the discussed studies; therefore, further studies could be useful in deeply investigating polyphenols’ effects in the adult population. Moreover, the use of only PBE and resveratrol represents an additional limitation: the implementation of this research area by using different polyphenols could provide further information and deepen the described scientific evidence for future use in clinical practice. Furthermore, the use of different psychodiagnostic tools for ADHD diagnosis might impact the robustness of the reported conclusions.

## Figures and Tables

**Table 1:** Risk of bias assessment.

	D1	D2	D3	D4	D5	Overall
Weyns, et al., 2022 [24]	+	+	+	+	+	+
Hsu, et al., 2021 [25]	+	-	+	+	+	-
Trebatická, et al., 2006 [26]	+	+	+	+	+	+
Tenenbaum, et al., 2002 [27]	+	+	+	-	+	+
Rafeiy-Torghabeh, et al., 2020 [28]	+	+	+	+	+	+
Weyns, et al., 2022 [29]	+	+	+	+	+	+
Dvořáková, et al., 2006 [30]	+	+	+	+	+	+
Dvořáková, et al., 2007 [31]	+	+	+	+	+	+
Chovanová, et al., 2006 [32]	+	+	+	+	+	+
Darzi, et al., 2022 [33]	x	-	+	+	+	-
**Domains** - D1: Bias arising from the randomization process. - D2: Bias due to deviations from the intended intervention. - D3: Bias due to missing outcome data. - D4: Bias in the measurement of the outcome. - D5: Bias in the selection of the reported results			**Evaluation** - x High risk of bias - - Some concerns - + Low risk of bias			

**Table 2:** Summary of studies investigating polyphenols’ efficacy on ADHD symptoms.

Study	Study Design	Number of Participants	Main Results
Weyns et al., 2022 [24]	Double-blind randomized clinical trial	88	Teachers ADHD Rating Scale after 10 weeks: ↓ score with PBE (26.07 to 12.61; *p* < 0.05 vs. placebo) Parents ADHD Rating Scale after 10 weeks: ↓ score with PBE (32.19 to 28.74; *p* < 0.05 vs. placebo) Teachers Social Emotional Questionnaire (hyperactivity subscore) after 10 weeks: ↓ score with PBE (14.25 to 9.00; *p* < 0.05 vs. placebo)
Hsu et al., 2021 [25]	Double-blinded randomized placebo-controlled cross-over study	20	Inattention sub-scale in Teacher SNAP-IV after 4 weeks: ↓ PBE group (91.4 to 73.4; *p* = 0.024 vs. placebo) Hyperactivity sub-scale in Teacher SNAP-IV after 4 weeks: ↓ PBE group (84.3 to 77.3; *p* = 0.024 vs. placebo)
Trebatická et al., 2006 [26]	Double-blinded randomized placebo-controlled study	61	CAP Teachers Rating Scale after 1 month of PBE administration vs. baseline and placebo: ↓ hyperactivity score (*p* = 0.008 and *p* = 0.044) ↓ inattention score (*p* = 0.00014 and *p* = 0.0067) Conner’s Teacher rating Scale after 1 month of treatment with PBE vs. baseline and placebo: ↓ inattention score (*p* = 0.07 and *p* = 0.049) Conner’s Parent rating Scale after 1 month of treatment with PBE: ↓ hyperactivity (*p* = 0.065 vs. placebo). Performance Scale: ↑ the Weight scores after 1 month of treatment with PBE vs. baseline and placebo (*p* = 0.019 and *p* = 0.05)
Rafeiy-Torghabeh et al., 2020 [28]	Double-blinded randomized placebo-controlled study.	66	Changes in parents’ ADHD-RS-IV scores: Total score in RVS group ↓ after 4 weeks (33.93 to 10.27) ↓ after 8 weeks (33.93 to 8.50) Time–treatment interaction *p* = 0.015 Inattention sub-core in RVS group ↓ after 4 weeks (16.33 to 5.47) ↓ after 8 weeks (16.33 to 4.50) Time–treatment interaction *p* = 0.032 Hyperactivity sub-score in RVS group ↓ after 4 weeks (17.60 to 4.80) ↓ after 8 weeks (17.60 to 4) Time–treatment interaction *p* = 0.036

Note: ↓ indicates a reduction; ↑ indicates an increase.

**Table 3:** Summary of studies investigating polyphenols’ efficacy in rebalancing oxidative stress pathways.

Study	Study Design	Number of Participants	Main Results
Hsu et al., 2021 [25]	Double-blinded randomized placebo-controlled cross-over study	20	GSH/GSSG ratio after 4 weeks of PBE supplementation vs. baseline and placebo: ↑ PBE group (*p* = 0.0358 and *p* = 0.0336) TBARS levels after 4 weeks of PBE supplementation: ↓ PBE group *p* = 0.0026 vs. placebo
Dvořáková et al., 2006 [30]	Double-blinded randomized placebo-controlled study	43	Biochemicals parameters after one month of PBE treatment: ↓ GSSG level (4.60 to 3.58 μmol/L; *p* = 0.013) ↑ GSH level (102.89 to 130.44 μmol/L; *p* = 0.0054) Biochemicals parameters after wash-out period: ↑ GSH levels (130.44 to 140.38 μmol/L; *p* = 0.007) ↑ TAS levels (1.02 to 1.09 mmol/L; *p* = 0.002)
Dvořáková et al., 2007 [31]	Double-blinded randomized placebo-controlled study	57	Dopamine in ADHD children after one month: ↓ in PBE group (226.1 to 202.4; *p* < 0.05) GSH/GSSG ratio in ADHD children after one month of treatment: ↑ PBE group (35.93 to 52.26; *p* = 0.05)
Chovanová et al., 2006 [32]	Double-blinded randomized placebo-controlled study	61	8-oxoG levels after 1 month of PBE administration vs. baseline and placebo: ↓ in PBE group (*p* = 0.012 and *p* = 0.014) ↑ TAS in ADHD children after the wash-out period from PBE (1.091 to 1.026; *p* = 0.0019)

Note: ↓ indicates a reduction; ↑ indicates an increase.

**Table 4:** Summary of studies investigating polyphenols’ efficacy in ADHD.

Study	Study Design	Number of Patients	Main Results
Darzi et al., 2022 [33]	Case-control study	200	Indirect relationship between dietary polyphenol consumption and the risk of ADHD (*p* < 0.001). The results remained significant after the adjustment for: energy intake BMI, socioeconomic status, age, and gender (*p* < 0.001)

## Data Availability

Not applicable.
